# Inhibitory activity of *Halobacillus trueperi* S61 and its active extracts on potato dry rot

**DOI:** 10.1080/21655979.2021.2024375

**Published:** 2022-02-15

**Authors:** Shuo Shen, Wei Li, Jian Wang

**Affiliations:** a Academy of Agriculture and Forestry, Qinghai University, Xining, Qinghai 810016 China; b Key Laboratory of Potato Breeding in Qinghai Province, Xining, Qinghai 810016 China; c State Key Laboratory of Plateau Ecology and Agriculture, Qinghai University, Xining, Qinghai 810016 China; d Key Laboratory of Qinghai Tibet Plateau Biotechnology, Ministry of Education Xining, Qinghai 810016 China; e Northwest Potato Engineering Research Center, Ministry of Education, Xining, Qinghai 810016 China

**Keywords:** Potato dry rot, *Halophilic bacteria* S61, inhibitory activity, biocontrol

## Abstract

This study investigated the inhibitory activity of *Halobacillus trueperi* S61 and its active extract on potato dry rot pathogens and aimed at contributing to biocontrol agent development during potato storage. Three kinds of pathogens were isolated as target pathogenic fungi from dry rot tubers and determined as *Fusarium acuminatum* (Qing 9A-2), *Fusarium equisetai* (Qing 9A-5-8) and *Fusarium tricinctum* (Qing 9A-1-1) by morphological and molecular identification. The strain *Halobacillus trueperi* S61 and its extract exhibited a higher inhibitory rate on both three pathogens (56.32–65.75 and 1.67–51.11%), notably the best suppression efficiency is presented in *Halobacillus trueperi* S61 and 40 mg/mL ethyl acetate extract. In terms of in vivo effects, both *Halobacillus trueperi* S61 and its ethyl acetate extract effectively reduced the decayed fruit and weight loss rate (0–20% and 7.59–16.56%) and enhanced the defensive enzymatic activities to improve resistance. In addition, strain S61 could be colonized on potato tubers, especially the highest amount of 1.55 × 10^7^ CFU/mL on fifth day for variety Xiazhai 65. Overall, *Halobacillus trueperi* S61 and its ethyl acetate extract could be considered as potential approach for biocontrol potato dry rot.

## GRAPHICAL ABSTRACT

**Figure:**
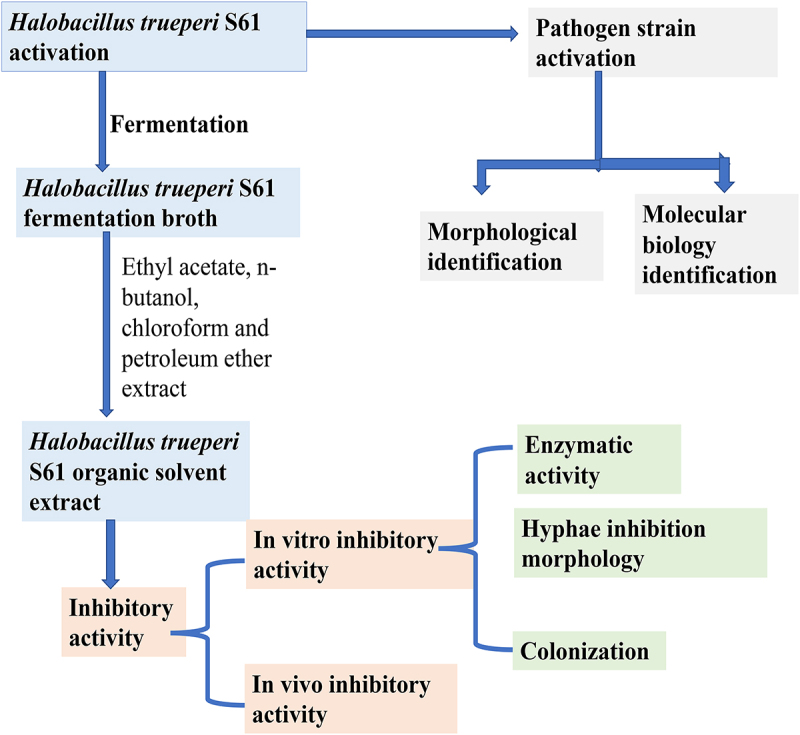

## 1. Introduction

Potato (*Solanum tuberosum L*.) as the fourth largest food crop and the main non-cereal crop, has contributed to food and nutrition security in developing countries [1]. The global potato cultivated area is 19.2 Mha with an annual production of 376.8 MT, of which 90.3 tons is produced in China [2]. However, potato crops are vulnerable to various phytopathogens further threatening quality and yield. Among them, post-harvest diseases induced by fungal pathogens cause significant economic losses worldwide [3,4]. For instance, early or late blight, *Verticillium* wilt, and powdery scab, especially dry rot as one kind of most serious postharvest diseases which causes 6%-60% yield loss annually and mainly infested by single or multi kinds of *Fusarium* [5]. There are reported 13 species associated with *Fusarium* dry rot worldwide [6] and Bojanowski et al. [7] isolated the predominant pathogens of *F. oxysporum* and *F. avenaceum* in China. The pathogen *Fusarium* capable resistance to low temperature and cause a high-level infectivity during potato storage. Apart from destroying tuber tissue, *Fusarium* also releases toxins such as fusaric acid accelerate transpiration and causes potato tubers to wilted, *enniatin* and *naphthazarins* disturb the energy supply, *zearalenone* damage the selective permeability of cell membranes and suppress the enzyme synthesis during plant growth [8–10]. After suffering from *Fusarium* dry rot, the potato surface appears necrotic wrinkles with brown to black, and the internal tissue infected by white, yellow or pink hyphae which cause shriveled, shrank and lesions, finally reduced the edible and economic value of potatoes [11].

Therefore, it is imperative to effectively prevent dry rot by agricultural, chemical and biological approaches [12–14]. Agricultural control is mainly focused on seed tubers selection and pretreatment, tillage approach, transportation or storage process. Chemical control is spraying chemical agents such as thiabendazole, fenpiclonil and thiabendazole, but it is easy to interfere with soil physicochemical properties then harmful to beneficial microbial metabolism and cause pathogens resistant [6,7]. Microbial biocontrol has attracted more and more attention due to environment-friendly and wide distribution, large number and complex diversity of metabolites of microorganisms. There are mainly through direct use of microbes or extract active ingredients for prevention and biocontrol pathogens. The mechanism of microbial control has been reported to be direct inhibited pathogens by biocontrol of primary and secondary metabolites as well as induce plants resistance [12]. At present, numerous endophytic microbials are effective for biocontrol plant diseases. The main strains for biocontrol potato dry rot are *Bacillus, Pseudomonas* and *Actinomycetes* [15]. Some studies have found that the spraying of *P. fluorescens* and *Enterobacter cloacae* could significantly inhibit the *Fusarium sambucinum* pathogen, notably *Bacillus* is outstanding in inhibiting fungi activity and promoting plant growth [16]. Mnif et al. [17] found that the extract of *Bacillus subtilis* could break hyphae and reduce the sporulation rate of *Fusarium*. Although, large number of active *Bacillus* have been found in extreme salt conditions such as *Bacillus rigiliprofundi 1MBB1T, Bacillus iranensis X5BT* and *Bacillus shacheensis HNA-14 T* [1], while only limited studies focus on the biocontrol of dry rot.

The *Halobacillus trueperi* isolated from extreme environments and has great potential to develop novel biocontrol agents due to stronger survivability and flexible adaptability. Additionally, dry rot pathogens widely occur in potato planting areas and their types varied with seasons, geographic locations and cultivars [18]. At present, there are few studies on inhibitory effects of moderately halophilic microbes on potato dry rot pathogen. Accordingly, this study identified the pathogens in three kinds of main potato types and detected pathogenicity, further evaluate the inhibitory activity of *Halobacillus trueperi* S61 and its organic solvent extracts (ethyl acetate, n-butanol, chloroform and petroleum ether extraction) on potato dry rot in vitro and vivo. The hypothesis that *Halobacillus trueperi* S61 enables to effectively inhibit *Fusarium* pathogens and aims at providing novel biocontrol approach for potato dry rot prevention.

## 2. Material and methods

### 2.1. Experimental materials

The potato varieties of Xiazhai 65, Qingshu 9 and Qingshu 2 were provided by the Institute of Biotechnology, Academy of Agriculture and Forestry Sciences (Qinghai University, China). The pathogens of Qing 9A-1-1, Qing 9A-5-8 and Qing 9A-2 were isolated from the diseased Qingshu 9 tuber, after purified then stored at 4°C. The strain *Halobacillus trueperi* S61 was collected from the Qrhan Salt Lake mud, then isolated and purified through the cultivation method. The potato dextrose agar (PDA) medium was made of 200 g glucose and potato, added distilled water to 1000 mL and adjusted pH to 7.2–7.4. The American type culture collection (ATCC) 213 medium comprised 10 g MgSO_4_ · 7H_2_O, 0.2g CaCl_2_ · 2H_2_O, 5 g KCl, 2.5 g peptone, 10 g yeast extract, 40 g NaCl and 12 g agar powder, then added distilled water to 1000 mL and adjusted pH to 7.2–7.4.

### 2.2. Pathogenicity determination and identification of potato dry rot pathogens

#### 2.2.1. Morphological and molecular biological identification of pathogens

The pathogens Qing 9A-1-1, Qing 9A-5-8 and Qing 9A-2 were activated and picked up the hyphae then inoculated on PDA medium plate by inoculation needle, further cultivated at 28°C for 7 days in constant temperature incubator (HZQ-F160A). Observe and record the substrate color, mycelial shape, and colony size from naked eyes, as well as observe mycelia and spore morphology under microscope, finally perform morphological identification according to the *Fusarium* identification manual.

For molecular biological identification, the mycelia of activated potato dry rot pathogens Qing 9A-1-1, Qing 9A-5-8 and Qing 9A-2 were scraped from the culture medium plate, then extracted deoxyribonucleic acid (DNA) according to Ezup column fungal genomic DNA extraction kit (Sangon Biotech, Shanghai, China) and performed polymerase chain reaction (PCR) amplification based on primers ITS1 and ITS4 (5′-CTTGGTCATTTAGAGGAAGTAA-3′ and 5′-TCCTCCGCTTATTGATATGC-3′). The amplification system was 2× PCR Mix 10 μL, primer 2 μL, DNA template 1 μL, added distilled water to volume 50 μL. The PCR reaction conditions included pre-denaturation at 95°C for 5 min, denaturation at 94°C for 30 s, annealing at 55°C for 30 s, extension at 72°C for 50 s, perform 35 cycles and repair extension at 72°C for 8 min. After the process, tested 5 μL PCR amplified product by agarose gel electrophoresis, then sent the recovered product to Sangon for sequencing. The obtained ITS sequence of pathogenic fungi was submitted to GenBank to obtain the accession number, then compared in the GenBank database of the United States Biotechnology Information Center. Finally, draw phylogenetic tree according to Neighbor-Joining method in MEGA 7.0 software.

#### 2.2.2. In vivo pathogenicity determination of pathogens

The concentration of the three pathogens suspensions Qing 9A-2, Qing 9A-1-1, and Qing 9A-5-8 was calculated using hemocytometer under microscope and added distilled water to adjusted 1 × 10^7^ CFU/mL by high-purity water machine (GREEN-Q3-20 T), then named as A, B and C, respectively. The single infection was inoculated with A, B and C individually, and the compound infection was inoculated with AB, AC, BC and ABC. Selected each variety of ten potatoes (Qingshu 9, Qingshu 2 and Xiazhai 65) with consistent size, free of disease and mechanical damage, the tubers were soaked in 2% sodium hypochlorite for 15 min and then disinfected with 70% ethanol. After natural drying, punch one hole in each tuber using an aseptic puncher (diameter 15 mm) and inject 100 μL single or compound infection pathogen suspension. After 7 days storage in the incubator at 28°C and 70%-80% relative humidity, the lesion diameter and decay were measured. According to the following formula to calculate the decayed fruit rate and weight loss rate:

1. Decayed fruit rate (%) = Decayed fruit number/Total fruit number
2. Weight loss rate (%) = (Weight before storage – Weight after storage)/Weight before storage×100%

### 2.3. Inhibitory activity of strain S61 and its extracts on potato dry rot pathogens

#### 2.3.1. Preparation of organic solvent extract from *Halobacillus trueperi* S61

The active strain S61 was inoculated into ATCC213 liquid medium and cultured in a shaker at 28°C 180 rpm for 7 days. Collected the fermentation broth and filtered through a Brinell funnel, then obtained filtrate and mixed with equal volume of ethyl acetate, petroleum ether, chloroform and n-butanol at 1:1, the mixture was suspended on rotary evaporator (EYELA N-1300) for three times to obtain strain S61 extracts.

***2.3.2 In vitro inhibitory activity of Halobacillus trueperi S61 and its extracts on potato dry rot pathogens***

The inhibitory effect of *Halobacillus trueperi* S61 on potato dry rot was determined by plate confrontation method. Cross petri dishes with marker and pour into PDA medium, then inoculated the activated *Halobacillus trueperi* S61 after cultivating in ATCC213 solid medium at 28°C. After 7 days cultivation, the pathogen diameter is measured and the inhibition rate of S61 on pathogens is calculated according to the following formula:

1. Inhibition rate (%) = (Control colony diameter-Treated colony diameter)/Control colony diameter × 100%

The *Halobacillus trueperi* S61 extract was prepared into 5, 10, 20 and 40 mg/mL, then 100 μL of the extract was dripped into Oxford cup and compared with distilled water with three replicates. According to the Oxford cup method, the culture dish was crossed with marker and poured into PDA medium, the sterilized Oxford cup was placed in the PDA medium at 2.5 cm from the medium center. *Fusarium* cultured for 7 d were cut by puncher (diameter 10 mm), and one part was placed in the center of each treatment plate. After incubation at 28°C for 4 d, the colony diameter of pathogens is measured and the inhibition rate is calculated.

### 2.4. In vivo inhibition effect of strain S61 and its extracts on potato dry rot pathogens

#### 2.4.1. Potato fruit treatment

Select disease-free potato tubers with consistent shape and size and soak 20 min in 5% sodium hypochlorite, after naturally dried then soaked in alcohol for 2 min, then punched one hole (diameter 1 cm) on the surface of the tuber after naturally dried.

#### 2.4.2. Electron microscopic observation of strain S61 ethyl acetate extract on pathogens

Sterilize petri dishes and pour into PDA, inoculated with Qing 9A-2, 9A-5-8 and 9A-1-1, then cultured in constant temperature incubator for 7 days. Fresh pathogen mycelia were picked and fixed in EP tube containing glutar-aldehyde fixative then sent to Kechuang Biological Company (Qingdao, China) for scanning electron microscope observation.

#### 2.4.3. In vivo inhibition of strain S61 and its ethyl acetate extract on potato dry rot pathogens

The pathogens group is inoculated 20 mL pathogen suspension in the hole, while the control 1 and 2 (CK1 and CK2) were inoculated with 20 mL distilled water and S61 suspension, control 3 and 4 (CK3 and CK4) were inoculated with 20 mL distilled water and 40 mg/mL S61 ethyl acetate extract. Cultivated in constant temperature incubator at 28°C and 60% relative humidity for 7, 14, 21 and 28 days, then measured weight loss rate and decayed fruit rate.

#### 2.4.4. Enzymatic activity determination

In the sterile environment, 1.0 g potato tubers were cut and put into the sterilized EP tube with a sterilized steel ball, and oscillated for 2 min in a 16,000-rpm shaker. The obtained frozen potato powder was fixed by adding phosphate buffer solution, then centrifuged at 5000 rpm and filtered 1 mL of supernatant for analysis. The enzyme activity of peroxidase, polyphenol oxidase, superoxide dismutase and catalase was determined by corresponding determination kit which was purchased from Jiancheng Biological Company (Nanjing, China).

### 2.5. Colonization of strain *Halobacillus trueperi S61* on potato tubers

#### 2.5.1. Strain *Halobacillus trueperi S61* cultivation and fermentation broth preparation

The *Halobacillus trueperi* S61 was activated on ATCC213 medium without antibiotics and cultured in a constant temperature incubator at 28°C until a single colony grew. The single colonies were picked up by burning inoculation needles and transferred to ATCC213 liquid medium, then cultured with shaking at 150 rpm and 28°C to obtain the strain S61 liquid culture medium.

#### 2.5.2. Inoculation of strain *Halobacillus trueperi S61* on potato tubers

The potato tubers were soaked in 2% sodium hypochlorite for 2 min, rinsed with distilled water and dried at room temperature, disinfected with 70% alcohol. The S61 liquid culture medium was collected and centrifuged at 4000 rpm for 5 min, 1 mL of distilled water was added after absorbing the supernatant, and adjusted S61 to OD600 of 0.1 then sprayed on the pretreated potato tubers.

#### 2.5.3 Colonization of strain *Halobacillus trueperi S61* on potato tubers

On the 1st, 2nd, 3rd and 4 to 7th day, 1 g potato was cut and placed in a sterile conical flask containing 10 mL 1% NaCl at 150 rpm for 30 min. Then, extraction of 100 μL diluted 10 times and spread in ATCC213 solid medium, cultivated 24 h in constant temperature incubator then counted the colonies number and calculated the colonization amount.

## 3. Result and discussion

In order to fully understand the pathogenesis of potato dry rot and develop possible biological control approaches, present study identified the dominant dry rot pathogens from three main varieties of potatoes through morphological and molecular analysis. Then, the *pathogenicity* through decayed fruit and weight loss as well as infection rate was evaluated. Importantly, in vitro and in vivo evaluated the inhibitory effect of *Halobacillus trueperi* S61 and its organic extracts on the potato dry rot pathogen and aimed at reveal the *Halobacillus trueperi* S61 biocontrol capacity and contribute to microbial biocontrol of dry rot.

### 3.1. Pathogenicity determination and identification of potato dry rot pathogens

#### 3.1.1. Morphological and biological identification of pathogens

**Figure 1.**
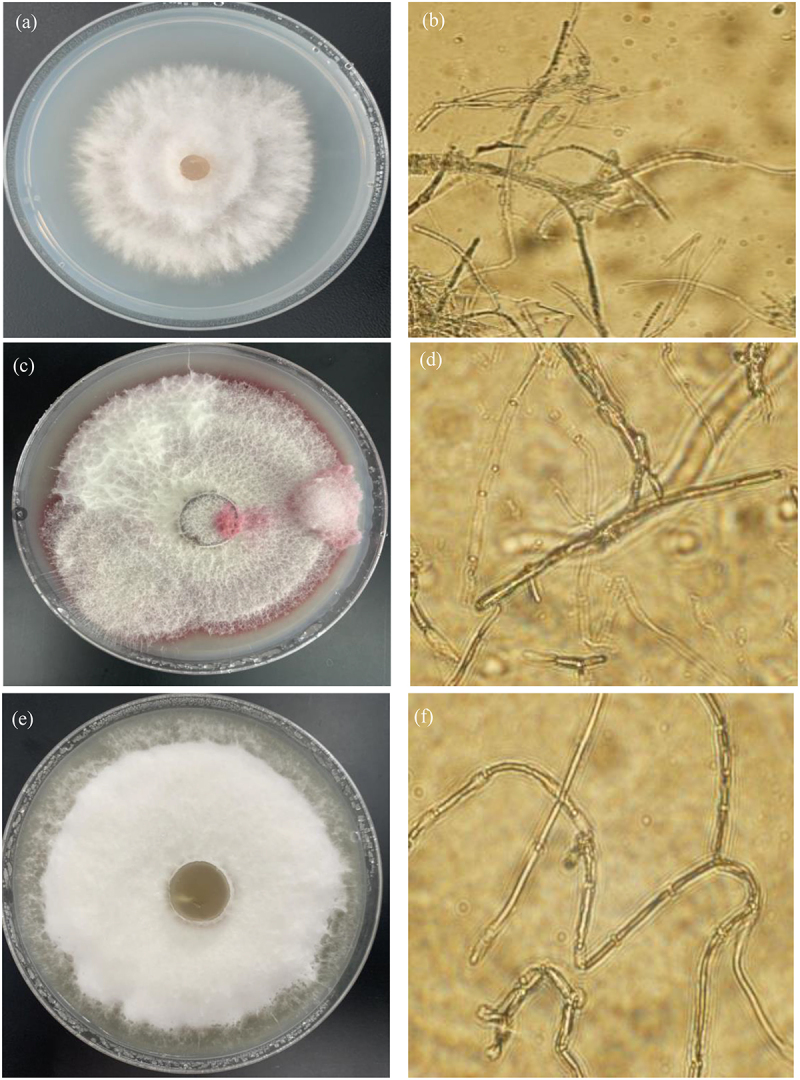
Morphological identification of Qing 9A-1-1 (a) on medium and (b) microscope (400), Qing 9A-2 (c) on medium and (d) microscope (400), and Qing A-5-8 (e) on medium and (f) microscope (400).

After pathogens cultivated in incubator at 28°C for 7 days, the characteristics of pathogen Qing 9A-1-1 were shown white hyphae with felt shape while non-discoloring of medium surface (Figure 1(a)), as well as the observed morphology under microscope was mostly rod-shaped hyphae with few branches and round spores (Figure 1(b)). Regarding Qing 9A-2 appeared felt shape hyphae with white and reddish color and the medium surface changed to red color (Figure 1(c)), as well as the observed morphology was rod-shaped hyphae with less branched and numerous oval and ovoid micro-conidia (Figure 1(d)). Concerning Qing 9A-5-8 was demonstrated white color hyphae with flocculent shape, and the culture medium showed yellowish color (Figure 1(e)), as well as the observed morphology was bent hyphae without branches, mostly septate and round or oval spores (Figure 1(f)). Similarly, Tiwari et al. [1] performed the identification of *Fusarium* dry rot based on morphology, and observed oval, elliptical and slender macro-conidia, white and brick orange mycelia, pale violet tinge and pale-yellow media. Bayona et al. [19] observed potato dry rot symptom with oval and kidney shaped micro-conidia, white and light purple aerial mycelium, as well as tan and light purple medium.

**Figure 2.**
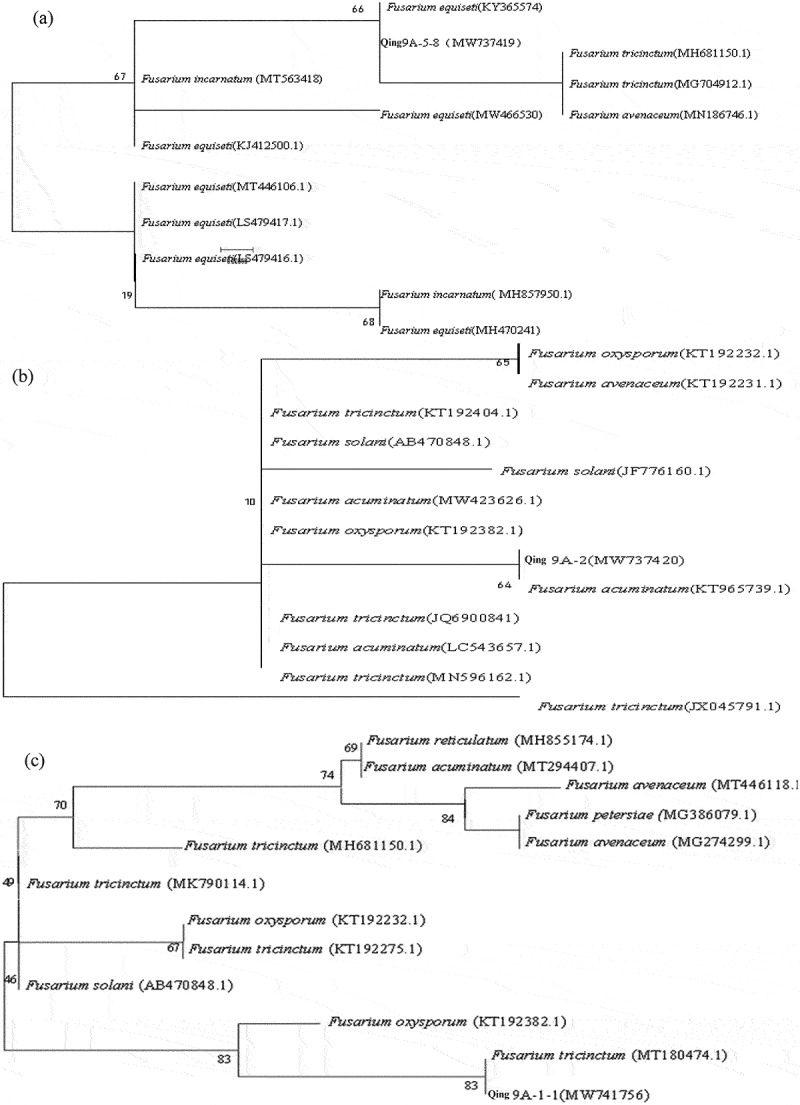
Phylogenetic tree based on sequences (a) Qing A-5-8, (b) Qing 9A-2 and (c) Qing 9A-1-1.

For identified the pathogens, ITS sequence of three pathogens were logged into GenBank database for BLAST retrieval, and the sequences with more than 98% similarity were selected from Genbank database for homology comparison, then build phylogenetic tree (Figure 2). The results demonstrated that the Qing 9A-5-8 (MW737419) and *Fusarium equiseti* (KY365574) gathered one branch (Figure 2(a)), Qing 9A-2 (MW737420) and *Fusarium acuminatum* (KT965739.1) clustered one branch (Figure 2(b)), Qing 9A-1-1 (MW741756) and *Fusarium tricinctum* (MT180474.1) gathered one branch (Figure 2(c)). Thus, three dominant pathogens of potato dry rot Qing 9A-5-8, Qing 9A-2 and Qing 9A-1-1 were affiliated to *Fusarium equiseti, Fusarium acuminatum* and *Fusarium tricinctum*. Schisler et al. [20] identified 14 kinds of main *Fusarium* causing potato dry rot and varied depending on geographic differences, among which *F. sambucinum, F. solani* and *F. avenaceum* are the dominant pathogens.

#### 3.1.2. Identification the pathogenicity of single and compound pathogens infection on potato

**Figure 3.**
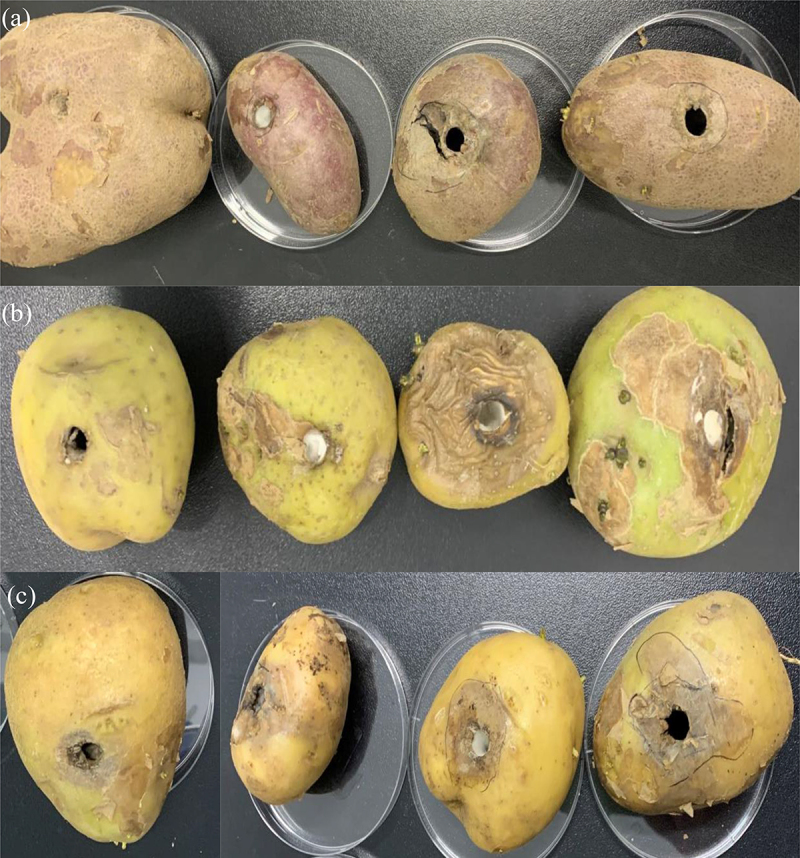
Pathogenicity effect of pathogens on potato in vivo. A was infection effect of pathogen on Qingshu 9, B was infection effect of pathogen on Qingshu 2, and C was infection effect of pathogen on Xiazhai 65. From left to right were infected by sterile water, Qing 9A-2, Qing 9A-1-1, Qing 9A-2 combine Qing 9A-1-1.

The single and compound infection of three pathogens on distinct variety of potatoes were evaluated (Figure 3). There were obvious shriveled or necrotic brown-black spots on the tuber surface of the three varieties, while the control was asymptomatic. The effect of single pathogens infect on Xiazhai 65 was greatest with the highest decayed fruit rate and weight loss rate (18.30–24.10 and 40–80%), while the lowest pathogenicity rate and weight loss rate identified in Qingshu 2 (9.70–18.70 and 0–20%). Notably the pathogenicity of Qing 9A-1-1 was greatest with 20–60% decayed fruit rate and 12.30–17.50% weight loss rate. Regarding compound pathogens infection, Qing 9A-2 and Qing 9A-1-1 were strongest with highest weight loss rate and decayed fruit rate (15.70–19.10 and 20–80%) as compared to other combinations (7.20–18.90 and 0–40%), the highest value appeared in Xiazhai 65 (9.4–19.1 and 20–80%) and lowest in Qingshu 2 (8.7–15.7 and 0–20%) (Table 1). Therefore, distinct potato varieties have different sensitivity responses to distinct pathogens, the pathogenicity of compound infection was lower than single infection which inconsistence with Tiwari et al. [1] who recorded the synergistic effect between *Fusarium spp*. and combined inoculation shows higher invasiveness than single inoculation. Maleki et al. [4] also pointed out that combined treatment was superior than individually in vitro and vivo condition.

**Table 1.** Effect of single and combine infection on three potato varieties

Variety	Qing 9A-1-1		Qing 9A-2		Qing 9A-5-8		AB		AC		BC		ABC	
	Weight loss rate	Decayed fruit rate	Weight loss rate	Decayed fruit rate	Weight loss rate	Decayed fruit rate	Weight loss rate	Decayed fruit rate	Weight loss rate	Decayed fruit rate	Weight loss rate	Decayed fruit rate	Weight loss rate	Decayed fruit rate
Qingshu 2	18.70%	20%	12.40%	20%	9.70%	0%	15.70%	20%	14.40%	20%	10.80%	0%	8.70%	0%
Xiazhai 65	24.10%	60%	20.90%	80%	18.30%	40%	19.10%	80%	18.90%	40%	9.40%	20%	9.90%	20%
Qingshu 9	17.50%	40%	16.60%	60%	12.30%	20%	17.50%	60%	14.60%	40%	9.30%	20%	7.20%	20%

A is Qing 9A-2, B is Qing 9A-1-1, and C is Qing 9A-5-8.

As for infection rate of single or compound pathogens demonstrated the fastest infection rate of Qing 9A-2 (83 mm/d), followed by Qing 9A-1-1 and Qing 9A-5-8 (58 and 44 mm/d) when infected on Xiazhai 65. For variety Qingshu 9, the fastest infection rate was 9A-2 (46 mm/d), followed by Qing 9A-1-1 and Qing 9A-5-8 (39 and 24 mm/d), while there is almost no infection on Qingshu 2. The strongest compound infection of Qing 9A-2 combined with Qing 9A-1-1 has highest infection rates on Xiazhai 65, Qingshu 9 and Qingshu 2 (83, 72 and 57 mm/d) (Table 2). Heltoft et al. [21] pointed out that *F. sambucinum, F. graminearum, F. solani* and *F. oxysporum* had severe lesions on most cultivars. Tiwari et al. [1] also proved higher infection of *F. sambucinum* than *F. oxysporum* (with lesion diameter 38–39.5 and 27.5–29 mm). Sana et al. [16] isolated 450 strains from potato and found Ar 10 shown the highest inhibition activity with 18 mm inhibited diameter. Khedher et al. [3] inoculated *F. solani, F. graminearum, F. sambucinum*, and *F. oxysporum* conidial suspensions on potato, observed clearly lesion diameter reduction (42.8–52.2%) after treated with *B. subtilis* V26. Xue et al. [8] proved the potato trichothecenes varied with distinct cultivars, *Fusarium* types and storage conditions. Overall, both three pathogens have a certain level infection on the three main variety potatoes, and the single infection ability was strongest in Qing A-2, followed by Qing 9A-1-1 and Qing 9A-5-8. Considering the pathogenicity and weight loss rate, the variety Xiazhai 65 was more susceptible than Qingshu 9 and Qingshu 2 to *Fusarium* dry rot *pathogens*, and compound inoculum *Fusarium acuminatum* and *Fusarium tricinctum* have stronger pathogenicity than individual infection.

**Table 2.** Infection rate of single infection on three potato varieties

Pathogen	Qingshu 9	Xiazhai 65	Qingshu 2
Qing 9A-2	Y = 0.46X+0.11	Y = 0.83X-0.73	-
Qing 9A-5-8	Y = 0.24X+0.45	Y = 0.44X+0.76	-
Qing 9A-1-1	Y = 0.39X-0.58	Y = 0.58X+0.43	-
AB	Y = 0.72X+0.63	Y = 0.83X-0.36	Y = 0.57X+0.12
AC	Y = 0.63X+0.45	Y = 0.74X+0.16	Y = 0.38X+0.62
BC	Y = 0.41X-0.53	Y = 0.34X+0.41	-
ABC	Y = 0.32X-0.27	Y = 2.28X-3.6	Y = 0.32X+0.81

A is Qing 9A-2, B is Qing 9A-1-1, and C is Qing 9A-5-8.

### 3.2. Inhibitory activity of Halobacillus trueperi S61 and extracts on potato dry rot pathogens

**Figure 4.**
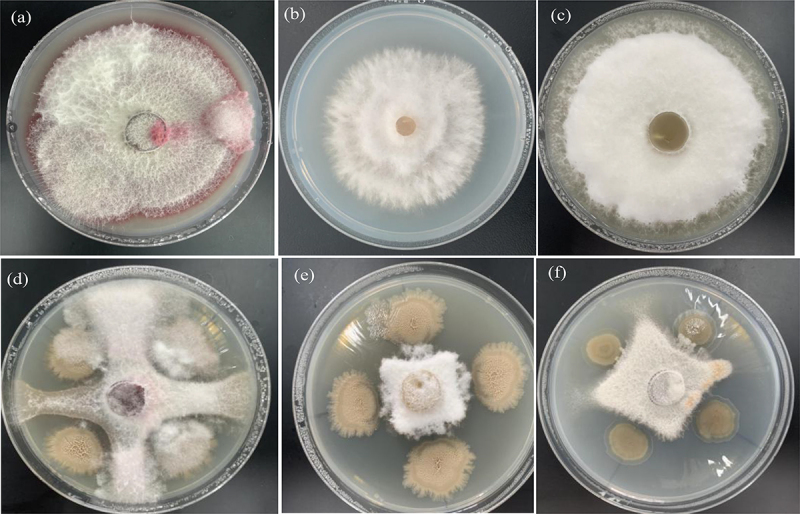
Inhibition of strain S61 (a) Qing 9A-2, (b) Qing 9A-1-1 and (c) Qing 9A-5-8 on three pathogenic bacteria in vitro, as well as confrontation result of Qing 9A-2 and S61 (d), Qing 9A-5-8 and S61 (e), Qing 9A-1-1 and S61 (f) on the medium.

The inhibitory rate of *Halobacillus trueperi* S61 and its extracts (ethyl acetate, n-butanol, chloroform and petroleum ether extracts prepared into 5.0, 10.0, 20.0 and 40.0 mg/mL concentrations) on pathogens were detected ((Table 3). Firstly, the pathogen diameter of *Halobacillus trueperi* S61 was significantly smaller (24.7–35.3 mm) and the inhibition rates of Qing 9A-1-1, Qing 9A-2 and Qing 9A-5-8 were 65.75%, 58.93% and 56.32%, which indicated the inhibitory activity of S61 on potato dry rot pathogens (Figure 4). Khedher et al. [3] reported the *Bacillus subtilis* V26 has obviously antifungal activity with reduced *Fusarium* diameter and mycelial (10.8–19.7%), and highest inhibition rate exhibited on *F. sambucinum* and lowest in *F. oxysporum* (85.3 and 54.7%). Kolaei et al. [5] pointed out that metabisulfite salts inhibited all selected fungi and 200 mM capable inhibit dry rot 100%.

**Table 3.** Inhibitory activity of strain S61 and its ethyl acetate extract on pathogens

		Qing 9A-1-1		Qing 9A-2		Qing 9A-5-8	
		Diameter/mm	Inhibitory rate/%	Diameter/mm	Inhibitory rate/%	Diameter/mm	Inhibitory rate/%
Strains	Concentration (mg/ml)						
16S strain	-	24.7 ± 2.7b	65.75%	32.0 ± 3.5ab	58.93%	35.3 ± 01.6a	56.32%
Ethyl acetate extract	5	21.0 ± 2.08a	30.00%	25.3 ± 1.49a	16.65%	25.6 ± 1.20a	14.44%
	10	19.0 ± 2.30b	36.67%	25.0 ± 1.52a	17.76%	22.3 ± 0.30b	25.65%
	20	17.3 ± 1.52c	42.20%	20.0 ± 0.50b	33.34%	21.3 ± 0.56b	28.98%
	40	14.6 ± 0.88d	51.11%	18.0 ± 1.12 c	40.00%	18.3 ± 0.86 c	38.89%
N-butanol extract	5	28.7 ± 0.23a	4.33%	29.0 ± 0.16a	3.30%	29.1 ± 0.08a	3.00%
	10	27.9 ± 0.41b	7.67%	28.1 ± 0.18b	6.30%	28.0 ± 0.57a	6.67%
	20	27.5 ± 0.25b	8.43%	28.5 ± 0.47b	5.00%	27.8 ± 0.38b	7.30%
	40	26.6 ± 0.21b	12.67%	27.1 ± 0.25 c	9.76%	27.5 ± 0.86b	8.33%
Chloroform extract	5	28.8 ± 1.79a	6.67%	28.92 ± 1.55b	3.67%	28.2 ± 1.96a	6.00%
	10	25.3 ± 0.95b	15.56%	27.4 ± 1.20 c	8.76%	28.7 ± 1.37a	4.33%
	20	24.3 ± 1.45 c	16.77%	26.6 ± 1.16a	11.34%	27.3 ± 0.96b	9.65%
	40	23.0 ± 0.65 c	23.33%	23.3 ± 1.18b	22.21%	26.7 ± 1.72b	11.34%
Petroleum ether extract	5	28.6 ± 0.60a	4.67%	28.2 ± 1.96a	6.00%	29.5 ± 1.49a	1.67%
	10	28.9 ± 1.51a	3.76%	28.1 ± 1.37a	6.33%	29.5 ± 1.62a	1.67%
	20	27.5 ± 1.41b	8.33%	27.6 ± 0.96b	8.00%	29.0 ± 0.70a	3.33%
	40	27.2 ± 1.81 c	11.00%	27.3 ± 1.72b	8.00%	28.8 ± 1.35b	6.67%
CK		30	-	30		30	-

Regarding S61 extracts, the inhibition rates of the three pathogens improved with the increased concentration (5.0–40.0 mg/mL). As to Qing 9A-1-1, the inhibition rate was 36.67–51.11% for ethyl acetate, 4.33–12.67% for n-butanol, 6.67–23.33% for chloroform, 4.67–11.00% for petroleum ether extracts. Regarding Qing 9A-2, the inhibition rate was 17.76–40.00% for ethyl acetate, 3.30–9.76% for n-butanol, 3.67–22.21% for chloroform and 6.00–8.00% for petroleum ether extracts. For Qing 9A-5-8, the inhibition rate was 25.65–38.89% for ethyl acetate, 3.00–8.33% for n-butanol, 6.00–11.34% for chloroform and 1.67–6.67% for petroleum ether extracts (Table 3). Notably, the S61 ethyl acetate extract has the highest inhibitory rate of 51.11%, 40.00% and 38.89% for three pathogens at the concentration of 40.0 mg/mL. As comparison, the S61 chloroform extract has higher inhibitory rate on Qing 9A-1-1 and Qing 9A-2 (23.33% and 22.21%) at the concentration of 40.0mg/mL while less effect on Qing 9A-5-8 (11.34%), while other extracts present lower inhibitory rate (3.00–12.67%) (Table 3). Ren et al. [7] compared distinct Black spruce extraction and proved ethyl acetate extraction has great antimicrobial potential. Elsherbiny et al. [9] perform methanol extraction of pomegranate peel demonstrated remarkable activity on *F. sambucinum* inhibition with 23.7–75.5% from 1.25–20 mg/ml concentration, greatest in 20 mg/ml to prevent potato dry rot. Kolaei et al. [5] identified there was no effect of organic solvents dichloromethane, chloroform, and ethyl acetate on antimicrobial activity while ethyl acetate extract has higher recovery and activity. The current study exhibited that *Halobacillus trueperi* S61 and its active extracts could inhibit the growth of pathogens, the ethyl acetate extract had the highest activity and the strongest inhibitory activity of S61 was acting on Qing 9A-1-1 (*Fusarium tricinctum*).

### 3.3. In vivo inhibitory effect of strain S61 and its extracts on potato dry rot pathogens

#### 3.3.1. In vivo inhibition of strain S61 and its ethyl acetate extract on pathogens

**Figure 5.**
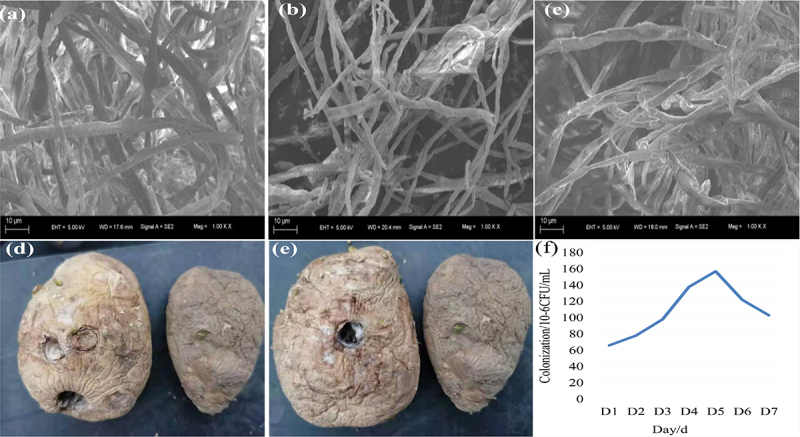
Electron microscopic observation of S61 on pathogens (a) Qing 9A-2, (b) Qing 9A-1-1 and (c) Qing A-5-8, as well as the in vivo effect of spraying S61 (d) and its ethyl acetate extract (e) on Qing 9A-2, and colonization of S61 on potato tubers (f).

Three pathogens were inhibited by *Halobacillus trueperi* S61 ethyl acetate extract, the hyphae all collapsed and shriveled at certain level and part of the hyphae was broken, and the growth point malformed and ultimate swelled (Figure 5(a-c)). Importantly, weight loss rate and decayed fruit rate are essential for potato quality during storage, water loss and pathogen infection during storage are vital factors for weight loss rate and strengthen with time, as well as the decayed fruit rate directly reflected the pathogens infection [22,23]. The weight loss rate of potato tubers was significantly reduced after inoculate S61 suspension (7.59%) and S61 ethyl acetate extract (7.63%) than single or compound infection (8.94–13.67% and 12.33–16.56%) (Table 4). After sprayed S61 suspension, the strongest single infection ability of Qing 9A-2 has 20% incidence and 13.6% weight loss rate, which were 60% and 8.3% lower than unsprayed S61 tubers. The compound infection of Qing 9A-2 and Qing 9A-1-1 had 10% incidence and 11.6% weight loss rate, which were 70% and 7.5% lower than unsprayed S61 tubers (Figure 5(d)). After sprayed S61 ethyl acetate extract, the incidence and weight loss rate of inoculated Qing 9A-2 were 20% and 13.5%, which were 60% and 7.4% lower than non-sprayed tubers.

**Table 4.** Control effect of spraying s61 suspension and its ethyl acetate extract

Treatment	Decayed fruit rate	Weight loss rate	Treatment	Decayed fruit rate	Weight loss rate
	16S strain			Ethyl acetate extract	
A	20%	13.67%	A	20%	16.56%
B	0%	9.89%	B	0%	10.32%
AB	10%	11.76%	AB	20%	15.47%
ABC	0%	8.49%	ABC	10%	12.33%
CK1	0%	7.23%	CK3	0%	8.89%
CK2	0%	7.59%	CK4	0%	7.63%

A is Qing 9A-2, B is Qing 9A-1-1, and C is Qing 9A-5-8, CK1 is inoculated sterile water, CK2 is inoculated S61 suspension, CK3 is inoculated sterile water, CK4 is inoculated S61 extract.

The compound infection of Qing 9A-2 and Qing 9A-1-1 had 20% incidence which was 60% lower than non-sprayed tubers, and 15.4% weight loss rates which similarly with control (Figure 5(e)). Khedher et al. [3] evaluated the effect of *B. subtilis* V26 on *F. solani, F. graminearum, F. sambucinum*, and *F. oxysporum*. Sana et al. [16] detected the antagonistic strains Ar10 treated 72 h shown positive effect on potato tubers protection with highest weight loss rate, necrosis and depth area (85 and 100%). Finally, inoculated *Halobacillus trueperi* S61 and ethyl acetate extract remarkably reduced weight loss rate and decayed fruit rate, three cultivars exhibited distinct efficacy in terms of pathogenicity and resistance activity, and the *Halobacillus trueperi* S61 demonstrated biocontrol capacity of dry rot pathogen infection.

#### 3.3.2. Response of enzymatic activity after sprayed strain S61 and *ethyl acetate* extract

**Figure 6.**
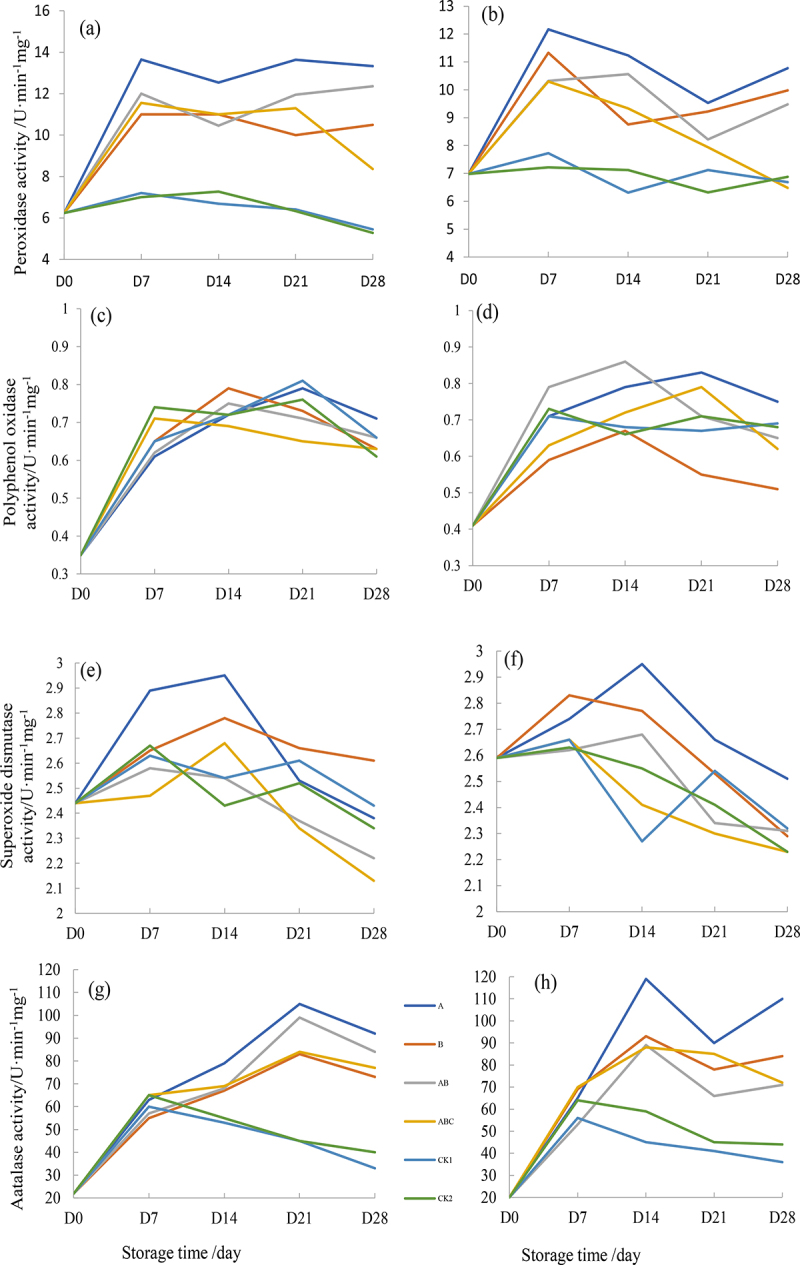
Response of enzymatic activity after sprayed strain S61 and its extract: peroxidase (POD) after spraying S61 (a) and its ethyl acetate extract (b), oxidase (PPO) after spraying S61 (c) and ethyl acetate extract (d), superoxide dismutase (SOD) after spraying S61 (e) and ethyl acetate extract (f), catalase (CAT) after spraying S61 (g) and ethyl acetate extract (h).

The biocontrol of soil-borne pathogens is related to antibiotics and hydrolytic enzymes, and the disease resistance of plants was closely associated with enzyme activity of the defense enzyme systems [3]. It is reported that beneficial microorganisms could activate the defense mechanism of plant reproductive system [24–26]. Peroxidase (POD) as one kind of plant defense enzyme systems and improved environmental resistance, and the activity was lower in control sprayed with S61 and ethyl acetate extraction (0.6 U·min^−1^mg^−1^). While the POD activity of sprayed with ethyl acetate extract was higher than S61 (7.3–12.2 and 7.1–12.1 U·min^−1^mg^−1^), indicated that the stronger bio-control effect of *Halobacillus trueperi* S61 (Figure 6(a-b)). Regarding to polyphenol oxidase (PPO) and superoxide dismutase (SOD) that enable to delay plant senescence and pathological changes, the activity continuously increased from 1–14 days (0.35–0.80 and 2.41–2.93 U·min^−1^mg^−1^), while the control earlier reached the peak value at 7 days (0.71 and 2.62 U·min^−1^mg^−1^) and then dropped down, which proved the S61 and its ethyl acetate extract strengthen the resistance to dry rot pathogens (Figure 6c-f)). Moreover, catalase (CAT) is capable of resisting the poison of hydrogen peroxide during the tuber infection process. The activity increased initially and achieved maximum value on 21 d (100 U·min^−1^mg^−1^) for S61, 14 d (120 U·min^−1^mg^−1^) for S61 ethyl acetate extract, while 7 d for control (60 U·min^−1^mg^−1^), which indicated that both S61 and its ethyl acetate extract were able to inhibit pathogens, and spraying S61 suspension was faster (Figure 6(g-h)).

Therefore, the change in defense enzyme activity associated with stress resistance and the increased activity effectively relieved the infection of potato dry rot pathogen. Similar phenomena also found by Al-Mughrabi et al. [22] who detected the increased activity of CAT, SOD and MDA when potato tubers were infected by potato dry rot. As reported, the *Fusarium* mainly produces variety of enzymes to degrade the cell wall, causing the potato tubers to corroded and further water loss and shriveled [27,28]. Khedhe et al. [3] proved that V26 is capable of producing cellulase, protease, pectinase and glucanase which could lyse some pathogenic fungi to promote plant growth and inhibit disease. *Bacillus* is a well-known bio-surfactant producer with broad-spectrum antibiotic activity and demonstrated significant antifungal and antiviral activities [16,29,30]. Khedher et al. [3] reported *Bacillus spp*. inhibited *F. sambucinum* and produced chitinases to protect potato tubers. Overall, the in vivo assay proved the antagonistic ability of *Halobacillus trueperi* S16 and its ethyl acetate extract on three kinds of *Fusarium* pathogens and sprayed S61 may have stronger competitiveness for nutrient and metabolic space which suppressed dry rot pathogens.

### 3.4. Colonization of Halobacillus trueperi S61 on potato tubers

Biocontrol agents based on bacteria and fungi have an ideal niche to control potato dry rot which attracted widespread attention [28,29]. Previous studies have found that *Bacillus amyloliquefaciens* could form good colonization and endogenous in maize, then used for biocontrol of maize leaf spot. The strains that can be colonize and endogenous in wheat are used for wheat scab/ head blight control [17–21]. In this study, *Halobacillus trueperi* S16 showed distinct activities on distinct kinds of *Fusarium* pathogens, which might be due to diverse factors such as biocontrol agent type or strain efficiency, pathogen type or invasiveness and the host susceptibility [31]. The number of *Halobacillus trueperi* S16 detected in potato showed an upward trend when spraying for 1 to 5 d, and the colonization of strain S61 in potato tuber reached the maximum of 1.55 × 10^7^ CFU/mL at 5 d. Thereafter, the colonization of strain S61 in potato tuber decreased during 5 to 7 days (Figure 5(f)). This indicated that *Halobacillus trueperi* S16 could form a certain amount of endogeneity and well colonized on Xiazhai 65 potato. In conclusion, the observation in vitro and vivo supported that *Halobacillus trueperi* S16 has great potential as a biocontrol agent for *Fusarium* potato dry rot.

## 4. Conclusion

The dominant pathogens of potato dry rot were *Fusarium acuminatum, Fusarium equiseti* and *Fusarium tricinctum*. Both pathogens have a certain level of pathogenicity on the three main variety potatoes, especially combine *Fusarium acuminatum* and *Fusarium tricinctum* has stronger pathogenicity than individual infection. The variety Xiazhai 65 was more susceptible than Qingshu 9 and Qingshu 2 to *Fusarium* dry rot. The *Halobacillus trueperi* S61 and its active extracts inhibited the growth of potato dry rot as well as enhanced the defense enzyme activity, notably the ethyl acetate extract had the highest activity and the strongest in vitro and vivo inhibitory activity of S61 was acting on Qing 9A-1-1. Overall, *Halobacillus trueperi* S61 is a promising novel biocontrol agent for *Fusarium* potato dry rot prevention.

## Highlight

- Dry rot pathogens dominant by *F. acuminatum, F. equiseti* and *F. tricinctum*.
- Variety Xiazhai 65 was most susceptible to *Fusarium* dry rot than Qingshu 9 and Qingshu 2.
- The strongest pathogen inhibitory activity of S61 was acting on Qing 9A-1-1 in vitro and vivo.
- The *Halobacillus trueperi* S61 and ethyl acetate extracts enhanced the defense enzyme activity.
- *Halobacillus trueperi* S61 was considered as potential approach for biocontrol potato dry rot.
